# Existential Displacement: Health Care and Embodied Un/Belonging of Irregular Migrants in Norway

**DOI:** 10.1007/s11013-020-09677-3

**Published:** 2020-05-25

**Authors:** Synnøve K. N. Bendixsen

**Affiliations:** Department of Social Anthropology, University of Bergen, Bergen, Norway

**Keywords:** Irregular migrants, Embodied unbelonging, Being-in-the-world, Violence, Healthcare, Governmentality

## Abstract

Drawing on fieldwork and interviews in Oslo and Bergen, Norway, this article discusses irregular migrants’ experiences of existential displacement and the tactics they use to try to re-establish a sense of emplacement and belonging. More specifically, it argues that irregular migrants’ experiences of embodied unbelonging are a consequence of a violent form of governmentality that includes specific laws, healthcare structures, and migration management rationalities. The article makes this argument by tracing how these experiences translate into embodied effects that feature prominently in migrants’ narratives of suffering while living in a country that purports to provide welfare services to all. The narratives of their state of being-in-the-world are ways through which migrants both experience and express the violence and deprivation they face. I argue that these narratives are instances of structures of feeling (Williams 1973), which are shaped by modes of governmentality. The article shows that irregular migrants’ coping strategies centrally involve faith, religious communities and friends. Irregular migrants draw on these relationships to get by, access healthcare, and to resist the (health) effects of social deprivation and political violence. These relationships allow irregular migrants to find meaningful ways of being-in-the-world and rebuilding, to some extent, a sense of entitlement and belonging.

## Introduction

This article explores the existential character of displacement and the ways in which irregular migrants struggle to find meaningful ways of being-in-the-world in Norway. It relies on an analysis of irregular migrants’ narratives about their everyday lives to examine the impact that being ‘illegal’ or irregular has on migrants’ well-being, health, and embodied belonging. The article uses the term ‘embodied un/belonging’ to call attention to the embodied, violent effects of governmentality and to migrants’ narratives of how they understand that process.

For more than a decade, critical migration scholars have shown that restrictive and inhospitable immigration contexts shape irregular migrants’ social and material conditions as well as their everyday psychosocial well-being and modes of “being-in-the-world” (Bloch et al. 2014; De Genova 2002; Ellis, Gonzales, and Rendón García 2018; Gonzales 2016; Gonzales and Chavez 2012; Willen 2007a, 2014).^1^ Migrant ‘illegality’ is a sociopolitical condition produced by the multiplication of everyday borders (Balibar 2004) and by “deportability” (De Genova 2002)—the process by which belonging is demarcated and exclusion generated. Embracing a phenomenological aspect of being irregular, Willen (2007b) advocates for the need to examine illegality not merely as a juridical status or a sociopolitical condition, but also as a mode of “being-in-the-world”. Such a phenomenological and bodily-aware approach aims to cast light on how irregularity also shapes a person’s lifeworld or being-in-the-world. I understand with Desjarlais and Willen (2007b) this as “a phenomenologically inclined account … which attends at once to the concerns and lifeworlds of [our ethnographic subjects] and to the interrelated social, discursive, and political forces that underpinned those concerns and lifeworlds” (Desjarlais 2005:369).

In what follows, I analyze irregular migrants’ narratives about their (lack of) well-being and being-in-the-world to show that ‘illegality’ is simultaneously a significant source of distress and structures their subjective experiences and lifeworld. I argue that the narratives are an expression of the everyday embodied conditions of being irregularized. As this article shows, the condition of illegality that marks irregular migrants’ being-in-the-world can create a shared structure of feeling (Williams 1977) that becomes a medium for the expression and management of their poor treatment and precarious situation. I view irregular migrants’ narratives of “embodied un/belonging” (see Mattes and Land introduction in this SI) as reflective of the broader political processes structuring irregularity, their living conditions, and the uncertainty of waiting. Their lack of access to health care and rights as irregular migrants are generated through politics and management and legitimized through political statements that as ‘illegals’ they do not belong in this nation-state. In effect, migrants experience an embodied unbelonging as expressed in the interviewees’ narratives. Thus, irregularity becomes an embodied disposition that is available to anthropologists as narratives. Looking at the ways in which being ‘illegalized’ shape migrants’ being-in-the-world and experiences of their well-being and health can shed light on the power of the state at its margins and how this power produces bodies that are physically present but ambiguously in/excluded in the nation-state.

Being categorized as an ‘irregular’ or ‘illegal’ migrant means having limited social and welfare rights, no access to the formal labor market, limited rights to healthcare services, and being ‘deportable’ (De Genova 2002). Migrants’ being-in-the-world is also shaped by their everyday practices and the institutions they interact with (Becker 2007; Horton 2004; Ong 1996), including those in the healthcare system. The current management of irregular migrants in Norway distinguishes between lives that are to be enhanced and lives that are not worth preserving (Das and Das 2007). This contributes to an exclusive politics of belonging (Yuval-Davis 2011) as it situates irregular migrants outside what Andrews (1999) calls the “political economy of hope”: the idea that in a free market economy, everyone can succeed under certain conditions.Though irregular migrants are socialized into irregularity (Bloch et al. 2014), they find ways of exercising various modes of agency and attain degrees of control and resistance (Bloch et al. 2014; Ellis and Rendón 2018; Bendixsen 2018) that allow them to re-establish a sense of belonging.

In the first part of the article, I discuss the health precariousness associated with being irregularized. I then present some of the narratives the irregular migrants I interviewed shared about their lack of well-being, unbelonging, and ways of being-in-the-world. In the third part of the article, I explore how migrants reconstitute a sense of embodied belonging and improve their well-being through everyday practices that allow them to reposition themselves as something more than only ‘illegal’ in Norway. In the final section of the article, I suggest that these narratives should be read as expressions of the harmful consequences of the government’s approach to dealing with irregular migration.

## The Embodied Character of Displacement and Unbelonging

Scholars have compellingly shown that irregular migrants’ social conditions are profoundly shaped by their illegal status in the society in which they live. Malkki (1995) suggests that refugees are subjected to the exclusionary nature of state sovereignty not only in relation to the state from which they fled, but also in relation to the one in which they apply for asylum. The correlation between living as an irregular migrant and poor health is well documented, for instance in the UK (Bloch, Sigona, and Zetter 2014) and the US (Gonzales 2016). In the US, studies have shown that the un/underinsured are also discouraged from using healthcare services, due to a payment system in which each medical visit increases one’s insurance costs (Becker 2007; Kim, Haney and Hutchinson 2012). This produces a specific way of being-in-the-world in which healthcare is felt as ‘not for us,’ which results in people becoming highly vulnerable to otherwise avoidable health crises.

De Genova (2002) focuses on the sociopolitical processes of ‘illegalization’ that produce the illegal status and its consequent sociospatial condition of deportability. Taking a critical phenomenological approach that accounts for how migrant illegality is configured and experienced, Willen (2007a, b) has shown that ‘illegality’ is a juridical status as well as a sociopolitical condition, and that migrants’ modes of being-in-the-world are powerfully influenced by local configurations of migrant ‘irregularity’ or ‘illegality.’ Migrants’ being-in-the-world includes their sense of embodiment and experience of time and space, both of which are strongly shaped by their ‘illegality.’ Clearly, racialized law and policy shape how the exclusionary mechanisms linked to their legal status affect irregular migrants. How and to what extent they do so depends on the particular nation-state’s regulations and laws, institutional practices, and public discourses on irregular migrants in the particular sociohistorical context.

Anthropologists have increasingly turned towards studying the various ways states seek to administer life and death (Das 2004). Research inspired by Foucault has shown how people are shaped and configured by biopolitics and governmentality. Governmentality is a form of governmental activity that seeks to guide and affect people’s conduct (Foucault 1991). Since the late 18th century, the state increasingly sought to control the intimate sphere of the population’s physical existence (Foucault 1994). This modern arrangement of power produced a form of biopolitical regime functioning at the micro level: the modern power should administer, regulate and improve the human body and body politics (Foucault 1994). Life became a matter of policy.

Biopower structures identities and generates actors as particular objects of control and regulations through a broad range of institutions, such as the church, educational and health care institutions and practices. Thus, biopolitics is part of a nation-building apparatus based on norms of inclusion and exclusion. Certain lifestyles, for example, are marked as culturally “inappropriate” and thus marginalized. Biopolitics configures and expresses a normative core of what is the “correct” way of life. Biopolitics is thus about rules of belonging and states of abandonment, as suggested in Giorgio Agamben′s (1998) idea of “bare lif”. It produces the borders of political communities (Makarychev and Yatsyk 2017), authenticating the essence of who should belong and who should not belong.

This article draws on Foucault’s perspectives on power to understand how the governmental management of life affects irregular migrants’ bodies and their way of being-in-the-world. Foucault’s theory of biopolitics casts light on the relationship between state policies and practices, public discourses, the conduct of state officials and healthcare personnel, and experiences of the body and being-in-the-world. Additionally, Agamben’s emphasis on the inclusion/exclusion paradigm and the conceptual distinction between physical life (‘zoe’) and political life (‘bios’)  provide an important starting point for reflections on the embodied un/belonging of irregular migrants. In Norway, as in other nation-states around the world, governmental policies, laws, and media representations of irregular migrants produce an image of irregular migrants as undeserving and undesirable political subjects (see also Briggs and Mantini-Briggs 2003).

This article provides an ethnographic case study of how biopower operates in relation to irregular migrants by focusing on the ways in which irregular migrants narrate their being-in-the-world and the formation of embodied un/belonging. It shows how the biopower induces and configures processes of exclusion/inclusion and embodied un/belonging that have detrimental consequences for people’s health and well-being (cf. Das and Poole 2004). Irregular migrants’ experiences of being stigmatized in the public sphere (by the media and politicians) and of social exclusion come to characterize their understanding of encounters with healthcare institutions (Bendixsen 2018) and generate a form of embodied unbelonging.

## Research Methods and Participants

It is estimated that there are between 18,100 and 56,000 irregular migrants in Norway (Mohn et al. 2014), a majority of whom are men. The data presented here are drawn from a larger ethnographic study of irregular migrants and their access to healthcare and political mobilization that I conducted in Norway from 2011 to 2014. My primary interest was not on “why they had left”, but rather exploring how irregular migrants understood their healthcare rights, what they did in case of illness and what consequences their lack of rights and living ‘illegally’ in Norway had on their lives.

Reason for leaving their “country of origin” is usually very complex and narratives about “why they left” and reasons for applying for asylum can change with time. Some, like the Afghan migrants, had left their “country of origin” for Iran years before they again left for Europe. Sometimes reasons for applying asylum might change during their journey to Europe or in Europe (due to, i.e., trafficking, violence, abuse, or religious conversion). As an anthropologists it brings along methodological and ethical issues to focus on the question “why did you leave”: migrants are used to receive this question from migrant authorities, police and journalists and thus many migrants have established a preset, coherent and consistent narrative on how to answer this question (Knudsen 1995). Attempting to distinguish whether or not someone is a “true” asylum seeker or a “labor migrant” as an anthropologist ignores that “anyone on the move may have a well-founded fear of persecution and be entitled to international protection” (Carling 2017). In this article, the migrants with whom I conducted fieldwork and interviews are all ‘irregular migrants’ who had applied for asylum but have been rejected and then overstayed the stipulated deadline for leaving the country. That said, ‘irregular migrant’ is a heterogeneous category in terms of gender, age, class, ethnicity, and religion. Furthermore, irregular migrants’ varying family statuses, education levels, language abilities, and social networks lead to stark differences in how they cope in the everyday.

I have conducted semi-structured interviews with more than 50 irregular migrants, the majority of whom were men between 20 and 45 years old. While I meet regularly some of these migrants over a period of 1.5 years during my fieldwork with irregular migrants protesting in the city of Oslo, others I meet only a few times, and yet others only once. The majority of the respondents were from Afghanistan, Iraqi-Kurdistan, Ethiopia, Eritrea, Iran, and Palestine. Like the majority of irregular migrants in Norway in the last two decades, most had become irregularized after their asylum application was rejected and they overstayed their deadline for leaving the country. Some irregular migrants leave the reception center before or after having received a rejection letter. Most of these migrants then move to Oslo both because they have a social network there and because as the largest city in Norway it has the most potential for informal work. Families frequently remain in reception centers because the high cost of living and poorly paid informal work make it very difficult to support a whole family in Norway. Single migrants tend to leave reception centers, in part because they fear being deported if they remain in government-run accommodation. While some of the irregular migrants I talked to had fled from war, violence and persecution, others had fled from economic poverty, family conflicts or personal challenges. During the talks and interviews, I did not explore the reasons for migrating and applying for asylum. While the migrant’s past experiences in their country of origin in all probability also shaped their experiences as being irregular in Norway, such aspects did not materialize during the conversations. What did appear as pertinent in shaping their being-in-the-world was aspects such as waiting for news of their (rejected) cases, being defined as ‘illegal’, and lack if regularized employment.

While I occasionally made use of a translator when speaking to the migrants, most of them spoke English or Norwegian. The interlocutors had been in Norway between 1 and 12 years by the time I interviewed them. In close cooperation with the board of the Healthcare Center for Paperless Immigrants (HCP), I approached many of the interviewees at the HCP in Oslo.^2^ I put up a description of the project on the noticeboard in the waiting room. When approaching people, I introduced myself as an anthropologist, explained my project, and asked whether we could meet somewhere else for an interview at a time and place of their choosing. The open-ended and semi-structured interviews lasted between 50 min and 2 h and explored their perception of their life, healthcare rights, and healthcare practices. Additionally, I did participant observation with irregular migrants who held a public protest in Oslo for 1.5 years.

I think of narratives as the stories people tell about themselves and their everyday lives and through which they can express forms of embodied belonging. The narratives in this article show that when combined, illegality, perceptions of well-being, and the constellations of problems in irregular migrants’ everyday lives present insurmountable obstacles to accessing healthcare services. Good and Good’s (1980:174–175) meaning-centered argument that “human illness is fundamentally semantic or meaningful” is particularly relevant in this regard. The narratives presented here do not primarily revolve around illness but around a lack of well-being and a troubled state of being-in-the-world. I focus on the manner in which these narratives recount personal and shared stories of social suffering, distress, and ultimately a lack of embodied belonging. They are clearly shaped by the migrants’ social and legal context and informed by their position in the world. Thus, in the interpretation of the data, I view the migrants as individuals belonging to a specific social world that generates specific ways of making sense of their pain and state of mind.

## Irregular Migrants and Healthcare in Norway

In Norway, the welfare system is particularly comprehensive and universal, in that social services are offered to all Norwegian citizens. Though there are fees (except for children under the age of 16), these are quite low, and specialized healthcare services, such as hospitals, are available at no cost (Søvig 2011). The Norwegian welfare state is promoted as providing care to the entire population, alleviating suffering, and making sure that no one lives in “undignified conditions” (Vike 2004). This ambitious way of taking responsibility for citizens’ health needs has been challenged in recent years as the previous perception of a simple correlation between citizens and the nation-state’s territory has been questioned. The rights of people who are not citizens and legal residents of the welfare state present a problem for a welfare state that has a moral goal of ensuring that none suffer within its territory (Bendixsen 2019). Asylum seekers, refugees, and people arriving on family immigration permits automatically have rights in the National Insurance Scheme once they are registered as resident in Norway. If an asylum seeker is rejected, and does not leave within the stipulated deadline for leaving the country, the person enters into a legal state in which he or she is irregularized.^3^ This is the category which is referred to in this article as ‘irregular migrant’. In turn, this means fewer social welfare and healthcare rights. Adult irregular migrants, defined as “people without legal residency in Norway” by the Directorate of Health, have the right to “emergency healthcare” as well as assessments of somatic and mental health issues within the primary and specialist healthcare services. They do have full access to shelters, but they do not have the right to specialized healthcare services and they might be asked to pay the costs of these services (Søvig 2011). Irregular migrants have quite extensive rights under the Act on Infectious Diseases (1994) in Norway (Søvig 2011), as in several other European countries. However, these rights to the treatment of infectious diseases (i.e., tuberculosis) are granted in order to safeguard Norwegian citizens from infectious patients, rather than because of a concern for the irregular patients’ health (Bendixsen 2019). In some European countries, such as Germany, health care workers were for a while expected to denounce irregular migrants, a practice which recollected the Nazi period and which healthcare workers frequently refused to follow (Castañeda 2013). Yet, in some cases healthcare professionals contact the police as a way of being loyal to law (Biswas et al. 2011). Frequently, NGOs and volunteers take up the responsibility to provide care to irregular migrants, with unintended consequences, such as inducing migrants to perform the role of the docile, thankful and undemanding patient (Huschke 2014).

Research on irregular migrant’s health shows that they have higher levels of mental and somatic illness due to deteriorating living conditions and high levels of stress (Hacker et al. 2015). In France, Larchanché (2012) has showed how the interaction among social stigmatization, precarious living conditions, and the climate of fear and suspicion caused by restrictive immigration policies deter irregular immigrants’ access to health care rights and, importantly, diminishes immigrants’ sense of entitlement to such rights. In the Netherlands, Hintjens et al. (2020), show that fear of the authorities, together with a lack of knowledge about rights or lack of support networks, contribute to that undocumented people avoid healthcare providers, unless it is an emergency. This worsens their health status. While in Denmark, Biswas et al. (2011) found that irregular migrants meet formal and informal obstacles in accessing the healthcare system, including limited medical rights, fear of being reported to the police, unpredictability in healthcare professionals’ approaches, poor language skills and lack of knowledge about the formal and informal healthcare system. Similar aspect characterized the migrants I interviewed in Norway. A majority of the migrants I interviewed were jobless or worked in the informal labor market, which is infamous for being precarious, under-paid, and dangerous. Experiences of ill health were often directly tied to migrants’ financial precarity and their dependence on the informal labor market. Having had their asylum application rejected, they had no legal right to work. Several studies quote migrants saying that “I cannot get sick” because they fear losing their job. In the absence of any social or institutional structures that would take care of them when ill, illness becomes an unbearable thought.

As in other European countries, and beyond, the responsibility for irregular migrants’ health has been taken on by voluntary organizations (Castañeda 2013, Willen 2011). In Norway, however, these are, perhaps paradoxically, partly funded by the Norwegian government. In the autumn of 2009, the Norwegian Red Cross and the Church City Mission (*Kirkens Bymisjon*) set up the Healthcare Center for Paperless Immigrants (HCP) at a secret address in Oslo and in 2014 another HCP was set up by the Norwegian Red Cross in Bergen. Run by volunteers, including medical doctors, nurses, and psychiatrists, these centers were founded after a study of ‘paperless’ migrants (Ottersen 2008) showed a need for such a service. When it opened, the HCP was controversial. The Norwegian government declared it “unwanted,” but decided it was not illegal to provide healthcare to people without a legal residence permit in Norway. In 2014, the right-wing Progress Party (FrP) suggested that the center’s practices were “illegal” and argued that it should be the duty of medical care personnel to report ‘illegal’ migrant patients to the police. As stated on the HPC website and in yearly reports, the ultimate aim of these institutions is to become superfluous and close down. Instead, in 2017, the centers saw 824 patients. By 2017, the centers had been used by 4200 patients from 116 different countries and had provided approximately 22,000 consultations (2009–2017).

Healthcare and social workers have always acted as gatekeepers, including in regard to the nation-state’s citizens (Ryyminen and Ludvigsen 2013). As a consequence of these laws and policies limiting healthcare rights and access, health service personnel unwittingly become accomplices in the migration management system. It is against the backdrop of these techniques of healthcare governance, which produce the social and health conditions of irregular migrants in Norway, that their particular narratives of being-in-the-world described below should be understood.

## Narrating Impaired Health and Destitution in a Space of Unbelonging

Embodied complications and negatively oriented states of mind were frequently expressed during discussions about what it meant to live ‘illegally’ in Norway. While sometimes concrete health problems were brought up, at other times more indeterminate health related problems were addressed. Sometimes migrants ascribed their embodied symptoms and responses to specific illnesses, at other times they did so less obviously. Headaches, stomach aches, pain, lack of appetite, mental torture, depression, becoming mad, and losing one’s mind featured as key manifestations of their ill health. For example, Abdel, a Palestinian in his mid-twenties who had been an irregular migrant for 1 year, had been part of a group of Palestinian irregular migrants who were involved in a public political demonstration in Oslo during my fieldwork. His co-demonstrators had told him that they should all leave Norway. “But where?” he wondered. “I get depressed by this. I don’t have any appetite.” Another example is Zoya, an Ethiopian woman who was in her early forties and had lived in Norway for 4 years. She talked about the problems she faced due to not having a valid identification document in Norway:

You cannot open a bank account. You cannot travel from one place to another. (…) So, you cannot integrate in society easily. So, we have so many problems. After that, after they [the Norwegian government] give us the second answer^4^, they just reject us, they just leave us. So, we are sandwiched. In our country there are problems, there is a dictator government. So, we cannot go back to our home. Here, we are denied, and we don’t get protection. We are in between. So, we are frustrated, we get mental torture, we are physically damaged. Some of our friends, some of our people, they got crazy, they become mad, some of them commit suicide. They cannot go to the society and integrate. They cannot go back to home. So, they are in between. If we are here, they know that, this government knows that they cannot send us to home because our government is not willing to accept us. They know that. (…). So, you just sit, you just sit (coughs) in your home, day by day. You are depressed and depressed and depressed. Some of the people don’t know that they are depressed because they just say, “I don’t want to see people,” “I don’t want to go there.” That’s a sign of depression. But nobody knows (coughs). After you go mad, they want to protect you. That’s stupid. After I lose my mind, if they give me gold or if they gave me anything, it’s meaningless for me. I need it when I’m healthy, when I can work, when I can do something. But after I am useless and mad, they will give me protection, it is meaningless. It’s nothing for me. (…) We are freely moving in the country, but we are prisoners, you know. We cannot go out of this country, because when we first came, we gave our handprints. So, I cannot go anywhere. They demand me here. I cannot go home. I cannot go anywhere. What can I do?

The policies and practices of excluding irregular migrants from rights tied to health and work induce particular expressions of distress. Zoya starts by talking about the practical difficulties of being an irregular migrant, but quickly moves to talking about the mental health effects. She relates her experience of being irregular by using expressions such as “mental torture,” “physically damaged,” “go crazy,” “suicide,” “in between,” “depressed,” “go mad,” “lose my mind,” and “useless and mad.” This deconstruction of her worth as a human being, and the detrimental consequences of how she experiences her own and others’ being-in-the-world are symptoms of impaired mental health and can simultaneously be understood as metaphors of violence and deprivation. The expressed emotional states cannot be disentangled from the associated physical states (cf. Jenkins and Valiente 1994). Zoya explicitly ascribes these feelings to her exposure to state-sponsored hostility. She also calls attention to how a deteriorated health situation can potentially improve her legal situation, trading off her mental sanity for her physical body: “you can get access if you go psychologically mad.” Zoya suggests that only when distress becomes visible as a disorder, does it ‘count’ and entitle her to treatment. In other words, distressed bodies and minds ironically start to belong when they manifest an identifiable disease/disorder.

## The Violence of Embodied Unbelonging

The living conditions in the reception center, characterized by waiting and uncertainty, also have embodied implications. Kofi, an Ethiopian man in his early thirties, talked about having left the reception center after 2 years to live as an ‘illegal’ migrant in Oslo city. He could not imagine returning to the center, even if life outside was hard. In the reception center, Kofi said,

there are many hundreds of Ethiopians living in different camps with no solution, from one year to ten years with no solution. So, these people are becoming psychologically victims, stressful, and some of them are not able to control their biological body even, their biological organs, they urinate on their body. That’s almost a psychological killing.

Here, Kofi frames people living in the reception center as unable to control their bodies, both mentally and physiologically. In other words, Kofi portrays the experience of being-in-the-world as that of an unbelonging with serious embodied consequences in the form of the loss of control over one’s body. This loss of bodily control, in Kofi’s account, is a form of violence caused by waiting without any solution in sight. His narrative further suggests a sense of recognition of and solidarity with others’ pain.

Chinua, an Eritrean in his early thirties, had back problems and had been transferred to a specialist by the reception center’s general practitioner. However, the specialist required him to pay. Since he did not have the money, the specialist asked the Norwegian Directorate of Immigration to pay, but the Directorate declined to do so. Chinua recalls:

“We don’t pay for him,” they said. Yeah. So, if you are sick, and if you cannot pay, you are nothing. (…) This is in Norway, in the civilized world. Heh? We are living in the 21^st^ century. (…). Nobody knows this situation, how we are suffering, how we are in trouble. But nobody knows us. Internally they kill us, that’s what. Just give up. If you are sick, I just pray to the Lord—please, Lord, just keep me [healthy], instead of becoming sick, you know. If you get sick, how tough it will be. You don’t have any ID, you don’t have nothing. You are just nothing. I can say, “you are nothing.”

Chinua’s narrative dramatically demonstrates that embodied unbelonging can be experienced as (internal) murder. Chinua and his family had decided to continue living in the reception center after they received the second rejection of their asylum application and a deadline for leaving Norway (usually 3 weeks after the second rejection). While this meant a higher risk of deportation, they could not afford anything else. They continued to receive a small income support at the reception center:

So, this money, you can imagine, how costly, how expensive it is in Norway, just to live, to eat meat, to buy some food. So, I’m forced to buy very cheap things, you know. (…) Sometimes I want to buy something for my baby. But I cannot afford. I cannot afford. I don’t have money; I don’t have any job. This gives me mentally, physically, internally, killing me. This is a silent way of killing people, you know. This is, you know, I can say that this is the last way of killing people. (…). Not by war, not by gun. A systematic way of killing—resources, mind, whatever. So, if you have paper, they treat you like a king, you know. They treat you like a human. If you do not have paper, you are like an animal. They don’t tell you this verbally. But you can see it in different ways.

I understand these embodied expressions as intimately linked to the family’s situation of not being accepted, provided with rights, or treated with dignity. Their bodies are deemed to be unworthy of thriving and are reduced to mere survival (Agamben 1998), which results in a challenging experience of embodied unbelonging.

The violence of being irregular that is so salient in Chinua’s narrative is equally striking in the descriptions given by Bilaal, a Palestinian in his twenties. During one of our many conversations, he explained that he felt he was outside rather than inside Norway:

Because I don’t have any kind of benefits or this kind of things that people have here or what the Norwegians say about Norway. I don’t have, I don’t see that I have my rights, I don’t have any kind of things. (…) It’s like in jail, in jail when you, when you are in jail and they want to torture you to get some kind of information from you. When they hit you, they don’t make marks on your body. So, if you go out, you don’t see that you are hit. And Norway is doing the same thing. They punch you in the face, but in a way that is not obvious. So, “we didn’t do anything, you are lying, we feed you, we give you food. What are you looking for?” See?

S: Yeah.

Bilaal: Welcome to Norge.

Bilaal’s emotional distress partially reflects the enormous discrepancy between how Norway is presented and how it treats him. His lack of control over his own life and the extent to which he is deprived of rights is remarkable. Though provided with the most basic necessities, he experiences his embodied exclusion as torture—a torture that leaves no physical traces. While the state is not letting him starve, it is also not letting him thrive. His “welcome to Norway” comment was made before the various movements of “welcoming refugees” started in several European countries in 2015. The irony in these words conveys not only a disappointment in how Norway treats him, but also a revelation of the true Norway as a country which, contrary to its self-representations, does not provide universal rights to everyone.

## Unbelonging on the Mind

Many migrants linked their (bad) health directly to their situation as irregular migrants, but also to past experiences of war, imprisonment, and poverty, and sometimes to previous health afflictions. For example, John suffered a head injury while doing military service in Eritrea. His health had deteriorated tremendously because he had slept on benches in Oslo parks during cold Nordic winters. At the time of this interview, John had been trying to avoid this:

John: I live with friends, one month with one, one month with another. But it’s not life. It’s difficult. Begging life. The worse problem is that your confidence is losing and losing and losing. Your mind is getting down, down, not getting up, up, up. That’s the most difficult for me. I can’t say, I’m not going to be like… because of the problem it’s eh… you know, things are not active, it’s going to be like down, down, down.

S: You think?

John: You are always busy with your mind; you are always busy. You are not free, you not being eh like smiling, you are not… eh, you are not free. You don’t feel free, ever again. You always worry. Because problem is not easy, to resolve that kinds of problem.

(…)

John: There are too much people like me here in Oslo, too much people. I was getting now a hope because I’m getting a normal [asylum] case. But I hope they will give me next month, I don’t know. My hope is to get the paper now. But too many people are like me. It’s very difficult. It’s almost like going mad (…), you can’t see me, but my mind is not right, not good. That’s the most problem. If someone does not have normal life, this is almost like being dead. If I’m not producing anything, I’m not doing anything, for example, I’m not old so I have to make a family, I have to make a life. (…) The mind is very difficult.

In John’s narrative, the condition of being irregular or ‘illegal’ produced various effects on “the mind” and health. Other interviewees made similar comments. John’s mind is always “busy,” he is “getting down,” and “losing confidence.” Being-in-the-world as an irregular migrant reduces life to a “begging life.” He recognizes that he is not the only person in this situation, thus acknowledging (while also perhaps making it clear to me) that the problems with his mind have its roots in a structurally induced situation rather than in a physiological disorder. John draws attention to the fact that while he appears healthy during our conversation, his irregular situation—i.e., being unable to marry, start a family or have a job, and facing an uncertain future—affects his mind. John talks about “going mad,” and that his mind is “not right,” and highlights his sense that his mind is trapped.

Like John, other irregular migrants talked about how their irregularity affected their mind and way of thinking, and some complained about being unable to focus or concentrate. Many described their irregular situation as a major source of uncertainty that created a psychologically difficult situation. Arman, a young man from Iran who had been in Norway for three and a half years, was very disappointed by how he was treated in Norway:

Arman: Like they changed completely my view about life. (…). A few months ago, for example, I had much stress, so I felt like some pain in my heart or it was really strange. But I knew it’s some hurting of the muscles from the hand. And it happened two or three times to me and then I said so it’s ok, I will not go to doctor. I was not sure if they are going to help me or not. So finally, because it became worse and worse, I’m talking about like four months ago. I didn’t sleep until morning, when I came to sleep out here [in the park]. And then they had some kind of test, and then they said ok, it’s because of stress. And then I said ok, what is the solution because I cannot sleep, like a few nights I did not sleep. I just sleep a few hours; I wake up again. And they said you have a problem because you are thinking a lot and you can… I said ok, so that’s why I’m sleepless also. I “said what can I do with it? Can I get to talk to a psychologist or to a special doctor for psychological problems?” They said “no, because you are illegal in Norway and that’s not possible. You need a special doctor [which we cannot provide you with].” So, I said “what is the solution?” She said “with what has happened to you, the best thing to do is to go and run in the forest.”

S: To go and run in the forest?

Arman: In the forest, yeah (laughs). I said “ok, like now it’s summer and maybe this is possible.” Can you imagine you sleep at three o’clock in the night and you wake up and you start running or walking in the forest or something? Funny actually.… and I told her “ok, now it’s summer you are telling me this. If it was in winter what could I do?” She said, “this is not my decision, I’m sorry but there is nothing that we can do for you.” So that’s it.

Arman describes sleeplessness and pain as partly caused by stress and thinking too much, processing his difficult situation repeatedly and incessantly, which makes it difficult for him to rest. Arman did not seek healthcare until he had insomnia and ongoing, long-lasting pain in the muscles of his hand. Like many others, Arman criticized the limited healthcare he received, being rejected by a psychiatrist, and only being told to be more physically active and spend time in nature. The latter is advice that Norwegian citizens also would receive from their general practitioner, as walking in nature is believed to relieve stress and reduce symptoms of depression. While Norwegian citizens would be familiar with such medical advice, to Arman it was culturally unfamiliar, and perhaps also somewhat demeaning.

## The Gendered Vulnerable Body

The irregular migrants’ marginalized and stigmatized position (Goffman 1963) is based on their lack of rights (to work, healthcare, and social justice), which limits their opportunities to pursue everyday social practices (such as going to work and taking vacations). This position is racialized and gendered. For example, one irregular Ethiopian migrant, Sarah, told me about the worst parts about her situation: “Especially, because I am a woman, things are hard on me. I have no right to live, to go to school, I’ve got nothing, this is the worst. And being a woman makes it more difficult.” When I asked her why she thought being a woman made her situation especially difficult, she responded, “If I have no money and don’t want to go to the main camp [reception center]—because I don’t want to remember the old times—maybe I’m forced to do something.”

Sarah was referring to sex work, but we were speaking through a male translator and he suggested that maybe she did not feel comfortable talking about this in his presence, so we decided to meet another time. At 31 years old, Sarah had been in Norway for 4 years and had been working until 8 months prior to our conversation. She lived with her sister in a small apartment close to the central railway station, and for 3 years she had been able to use official documents she received due to a bureaucratic error to work. However, by the time of our conversation, she had “no job, no nothing” because the bureaucracy had discovered the error and stopped issuing the documents. Sarah’s comments point to a bodily vulnerability, an embodied un/belonging that is gendered. While her body might help her survive, she feared this solution (that she might be pushed into sex work), which made her situation even more unsettling. Her body thus was a potential commodity that could provide a means for survival. At the same time, in her account, exchanging sex for access to economic resources was an option she only contemplated as the direct result of structural violence.

As these migrants’ narratives show, unbelonging can create particular forms of embodied dispositions and expressions. However, the migrants were not helpless in their search for alternative pathways to health, well-being, and belonging.

## Reinstituting Embodied Belonging Through Substitute Healthcare Services, Religiosity, and Friendship

While irregular migrants cannot resolve the uncertain situation that makes them “go crazy,” they find some relief for their poor physical health and troubled state of mind by visiting their general practitioner and the HCP. Some sought out physicians with names thought to indicate an immigrant background, as the irregular migrants believed these physicians would be less likely to notify the authorities and more likely to understand their particular situation.

In contrast to those who did not visit any healthcare services, including Emergency Rooms, other irregular migrants went to the HCP frequently even when they had no symptoms of physical illness. The healthcare volunteers believed this to be connected to a fear of becoming ill. But irregular migrants also visited the HCP because it was a social and safe place where they could have free tea and socialize with Norwegians and other irregular migrants. For many irregular migrants it represented a welcoming space within an otherwise antagonistic society (cf. Willen 2014) and a way through which they could constitute embodied belonging.

Belonging and well-being were also reconstituted through religious practices and spending time with friends.

Chinua: So, you understand, sometimes you fight with people about ideas. (…) I am tired of fighting with people, just ignore it. So, the only thing what I can do that time is praying to the Lord, God makes change, God is heaven. God, he is our leader – this is my belief as a Christian. I’m a born-again Christian. And I’m a church leader in the Ethiopian committee in Bergen.

S: Ah, in Bergen? Really?

Chinua: Yeah. Preaching the gospel to the people, in my language. So this is the only thing I can do these days. I don’t have nothing. I’m just, every Saturday we have a church meeting here in Pinsekirken [Pentecostal Church].

For Chinua, as for a number of other irregular migrants of different faiths (predominantly Christian and Muslim), visiting the church, mosque, or other religious spaces was a way to ease their mind, feel at home, and reconstitute belonging. Sometimes the religious community would pray for their health and for their papers. Praying and going to church or the mosque regularly also gave the otherwise monotonous week direction, variation, and a sense of time and place. Many migrants, including Chinua, perceived the religious space as enabling them to regain their sense of dignity and restore human equality in front of God. Religious sentiments and practices also fostered their hope for change. After having experienced a loss of control, leaving their lives to a higher, holy power able to bring about a change for the better was a remedy.

Other migrants talked specifically about the social support they received from friends as a source of well-being. Spending time with friends contributed to a reconstitution of their sense of self that was not tied to being an irregular migrant. Several migrants told me that they deliberately spent time with friends in order to forget their physical, psychological, emotional, and/or spiritual pain. John, for example, an Ethiopian in his early thirties, called upon his friends regularly. Having lived in Norway for 2 years, he was worried that the police would deport him. Thus, when he received the first rejection of his asylum application, he had decided to leave the reception center. He called his friends from Ethiopia who lived in Oslo and told them about his problems. He explained:

Then I went out, I see the people, I tried to gather those people from my home country. I stayed with them. And then we get friends, old friends, that we can’t stay without. Old friends are good to you, because a friend is a friend, you know him, he knows you, he doesn’t have chance to leave you. And then after a while I can get small job, like moving from place to place, from home, like that. Still I live like that.

Research indicates that irregular migrants may have ambiguous relationships with friends and co-ethnics due to the uneven power dynamic that exposes irregular migrants to exploitation in the informal labor market (frequently recruited through friendships) and ‘survival sex’ (Bendixsen 2018, Kjærre 2010). However, in the above quote John describes his friends as representing the necessary help and shelter he needed for survival, both in terms of material necessities and emotional and embodied belonging. John had stayed in a reception center when he first arrived to Norway in 2006.

John: Yes, I stayed there around five or six months. I was a little depressed. I was very active in Ethiopia. I was engaged in different kinds of activities. When I go to the camp [reception center] and I stay there I became depressed. So immediately I said to myself, if I stay like two months or three months here, I’m going to be sick. So, what I pray for, I just go out to Oslo to live with my friends. And then finally I arranged everything.

S: So, you had friends in Oslo who could help you?

John: Yeah, you know, not friends, Ethiopians. You know, in our society if you are Ethiopian you are going to be responsible. I just call you and come to your house, can you give me two days, three days shelter? They say to me ‘yes’. This is Ethiopian culture. That is what I did.

S: I understand. And… Did you know anyone before you came to Norway?

John: No. I was a lot of places in Europe. I was in Spain, Greece, Italy, a lot of places. But here in Norway, it’s my first time.

John had been afraid that the police would stop him on the street:

John: I was scared, because if they take me, they will send me to Italy. And then I will be facing a new problem. And immediately I will be dying, or I will be sick, I worry about that.

S: Did that prevent you from going outside, did you go on the street?

John: Yeah, but I have to because I need to be sometimes relaxed. I can’t stay alone. I can’t live alone at home, I can’t live alone. If I am alone, I will be stressed. So, I have to be close to people, I have to be close to people, to talk, to be relaxed. Without that it will be a problem.

While being engaged in religious activities and bonding with friends engendered feelings of inclusion and belonging, the ‘forgotten’ object of being irregularized does not disappear but is pushed out of consciousness for a moment (Darghouth et al. 2006). Thus, while social interaction is not a cure for the pain, it opens up another course of action and a space where the interviewees describe being distracted from anxiety and pain (cf. Darghouth et al. 2006). Socializing enables migrants to feel ‘relaxed’ and to belong to a welcoming religious or ethnic community. Being close to others, talking, laughing, and resting, bring calmness and tranquility which helps them forget their painful and worrisome situation at least for a while. This calls attention to their need to belong to a community with others in order to deal with their precarious situation. Furthermore, religious practice, whether pursued in a group or individually, constituted a remedy for their pain through which they could regain a sense of a meaningful life and the hope that it would improve in the future.

## Discussion and Conclusion

Governmental practices, health regulations, and public discourses that construct irregular migrants as undeserving subjects, potential criminals, and non-taxpayers, significantly shape irregular migrants’ being-in-the-world inasmuch as it contributes to their sense of embodied unbelonging. Facing a situation in which they have no rights to healthcare services or an identity number, cannot afford to pay for healthcare, and live in constant fear of the police and deportation profoundly affects the way irregular migrants understand their own life, their body, and their illness(es). The healthcare barriers they are confronted with include not only their limited legal rights but also a particular form of being-in-the-world as disenfranchised from the right to live a healthy life.

Health regulations and practices become part of migration control and contribute to a mode of governance that works through exclusion and willful neglect. This ultimately produces specific subjects that are viewed as undeserving of care and discouraged from seeking essential treatment (see also Horton 2007). The healthcare available to those living in Norway ‘illegally’ is reduced to that provided by charities. This leads to people delaying treatment, the deterioration of their health conditions, and self-exclusion from the healthcare system. In this sense the irregular migrants themselves contribute to their own subjectification as ‘illegal’ bodies without any rights in Norway.

This differentiated healthcare system is not an inconsistent exception in an otherwise well-organized welfare system. Instead, it is a technique of managing irregular migration, both in relation to those who have arrived in Norway and in relation to those who have not (yet) arrived. As part of this technique of containment, the healthcare system inadvertently teaches irregular migrants to suspend care and treatment by conveying the message that they are not welcome. In this sense, healthcare provision becomes an essential part of the larger ‘illegalization process’ of irregular migrants in Norway.

The present study has looked at how irregular migrants’ narratives of their everyday life revolve around troubles and pain that are not conceptually limited to the boundaries of the individual body. I understand the narratives about their minds “going mad” or “not working” as expressions of experiences of social suffering, being treated unfairly, and of an anxious anticipation of the future. In their narratives, symptoms of illness or ill-being included insomnia, lack of appetite, despair, and sadness. Such feelings are explicitly connected to their exposure to state-induced structural violence and their own specific legal and social condition. The migrants speak of both personal and interpersonal spheres of existence and suffering (cf. Darghouth et al. 2006). Their irregularity comes with severe life challenges and social, economic, and familial stressors that cause pain and illness. Insomnia, feeling down or mad, and depression emerge as the embodied expressions of an unjust world of violence and deprivation.

I suggest that while some of these narratives refer to health symptoms, at times the narratives about pain and illness are ways through which irregular migrants both experience and express their social distress. When expressing their state of being-in-the-world as irregular, all of the migrants talked about their bodily state, including how their mind was (not) working. The meaning they ascribed to their irregularity emerged from both individual and shared dimensions of their experiences of irregularity. The migrants’ being-in-the-world was defined by anxiety about not having a home or any prospect of an income in the near future, as well as by their anxieties in regard to their own and their children’s future. Their experience of unbelonging was also embodied in that they did not have the right to healthcare services (beyond emergency treatment) or to bodily well-being. This embodied unbelonging was narrated through references to how their state of mind was negatively affected. This becomes an instance of what Darghouth et al. (2006) have described as the potential of the body to step over the boundaries of verbal language. In a literal and metaphorical sense, the body can become a medium for expressions of personal and social distress.

I suggest that the expressions the migrants used to convey their disturbed state of mind are not only a description of the state of their well-being but also a response to the sense of deprivation “when language seems to fail” (Das 1996:70). These expressions are instances of what Raymond Williams (1973) called structures of feeling, which result from the power and relations of production predominating in a specific historical era and location. A structure of feeling is a hegemonic cultural configuration that validates and systematizes these relations (Williams 1973). These structures are not static, but can change in relation to fundamental discontinuities, such as those deriving from irregular migration.

The narratives presented here suggest that the modes of governmentality that target the irregular migrant population also shape the emotions of that marginalized group. The stories' individuals tell about their states of mind powerfully communicate their experience of irregularity. They also show that emotions can be a component of embodied experience. Drawing on Jenkins and Valiente (1994), I argue that the ways in which the migrants express their being-in-the-world (using expressions such as sad, going mad, and down) cannot be untangled from their related physical states. The migrants narrate their being-in-the-world as embodied responses to and outcomes of a world of inequality, injustice, and dehumanization.

Everyday life as an irregular migrant brings forth situations that unsettle social relations, reduce trust at the individual level and in the asylum system (Bendixsen, Kjærre, and Ytre-Arne 2014), and create multiple stressors that come to weigh heavily on migrants’ desires, beliefs, hopes, and fears. Thus, this study is also an exploration of how pain is framed within a larger framework of social distress.

The state’s approach to controlling irregular migration through limiting healthcare and welfare rights and thus creating extreme health inequalities is a form of structural violence that emerges as a central theme in the migrants’ narratives. Socioeconomic exclusion creates social hierarchies, both in terms of legal status and financially. The stories about varied forms of ill-being have metaphorical traits and, consciously or unconsciously, they feature a specific language about suffering. The migrants’ negative state of mind exists both as a confirmation and a defiance of life as irregular migrants: as a confirmation, its configuration reflects the tearing away of belonging and ‘normal life,’ an embodied testimony to their larger social and legal position. As defiance, it can be considered as a rejection or even resistance to governance practices that render migrants non-deserving subjects.

Irregular migrants also make use of various tactics of survival and ways to maintain hope for a better future, such as seeking recognition and inclusion from religious communities and friends. Being with friends and/or pursuing religious practices represent both internal and external sources of resilience that they can draw on to counter their sense of marginalization, uncertainty, and loss of control over their lives.

Finally, the irregular migrants’ narratives attest to the Foucauldian thesis that in modernity violence is redirected: institutional practices have transformed the nature of premodern punishment into a more deceptive and pervasive ordering of bodies. It shows that healthcare rights have become a technique of governing populations and producing particular kinds of subjects (Dean 1999). It is an empirical description of the disciplinary turn in which, in modern societies, inequality is biologized (transcribed into the body) rather than ritualized (inscribed onto the body) (Fassin 1996). The narratives of embodied unbelonging constitute a political expression of the state of the world (Fassin 2008:534). Furthermore, the narratives express the everyday violence that Walter Benjamin (1927) described as violence that is not seen as violence and illustrate its role in producing human anguish and distress. The insular nature of the migrant’s being-in-the-world comes to stand for a collapse of a social world where uncertain waiting is ingrained in an irregular condition and hopes for a future are constrained.

## Footnotes

1 The concept being-in-the-world originated in the discipline of philosophy with phenomenologists such as Maurice Merleau-Ponty and Edmund Husserl.

2 Personal data were collected based on informed consent and were managed in accordance with the Norwegian Personal Data Act, including General Data Protection Regulation, and University of Bergen’s regulations.

3 Labor migrants who are working without legal permission in Norway can also be termed irregular migrants. Irregular labor migrants will usually not have applied for asylum, and thus not have been in contact with the asylum system and the rights pertaining for the category “asylum seeker”. Whether or not one has applied for asylum creates a different situation as an irregular migrant. For example, rejected asylum seekers who are irregular are allowed to stay in regular reception centers even if the financial support a person would receive in the center is regulated according to the person’s legal status.

4 In Norway, if an application for protection has been rejected (negative decision), all asylum seekers have the possibility to be assigned a lawyer by the UDI and can appeal the decision. If the UDI does not reverse its decision, it will send the case to the Immigration Appeals Board (UNE).
